# Targeting multiple myeloma with nanobody-based heavy chain antibodies, bispecific killer cell engagers, chimeric antigen receptors, and nanobody-displaying AAV vectors

**DOI:** 10.3389/fimmu.2022.1005800

**Published:** 2022-11-02

**Authors:** Julia Hambach, Anna Marei Mann, Peter Bannas, Friedrich Koch-Nolte

**Affiliations:** ^1^ Institute of Immunology, University Medical Center Hamburg-Eppendorf, Hamburg, Germany; ^2^ Department of Diagnostic and Interventional Radiology and Nuclear Medicine, University Medical Center Hamburg-Eppendorf, Hamburg, Germany

**Keywords:** nanobody, multiple myeloma, CAR, heavy chain antibodies, BiKE, AAV, VHH, CD38

## Abstract

Nanobodies are well suited for constructing biologics due to their high solubility. We generated nanobodies directed against CD38, a tumor marker that is overexpressed by multiple myeloma and other hematological malignancies. We then used these CD38-specific nanobodies to construct heavy chain antibodies, bispecific killer cell engagers (BiKEs), chimeric antigen receptor (CAR)-NK cells, and nanobody-displaying AAV vectors. Here we review the utility of these nanobody-based constructs to specifically and effectively target CD38-expressing myeloma cells. The promising results of our preclinical studies warrant further clinical studies to evaluate the potential of these CD38-specific nanobody-based constructs for treatment of multiple myeloma.

## Introduction

**Figure 1:**
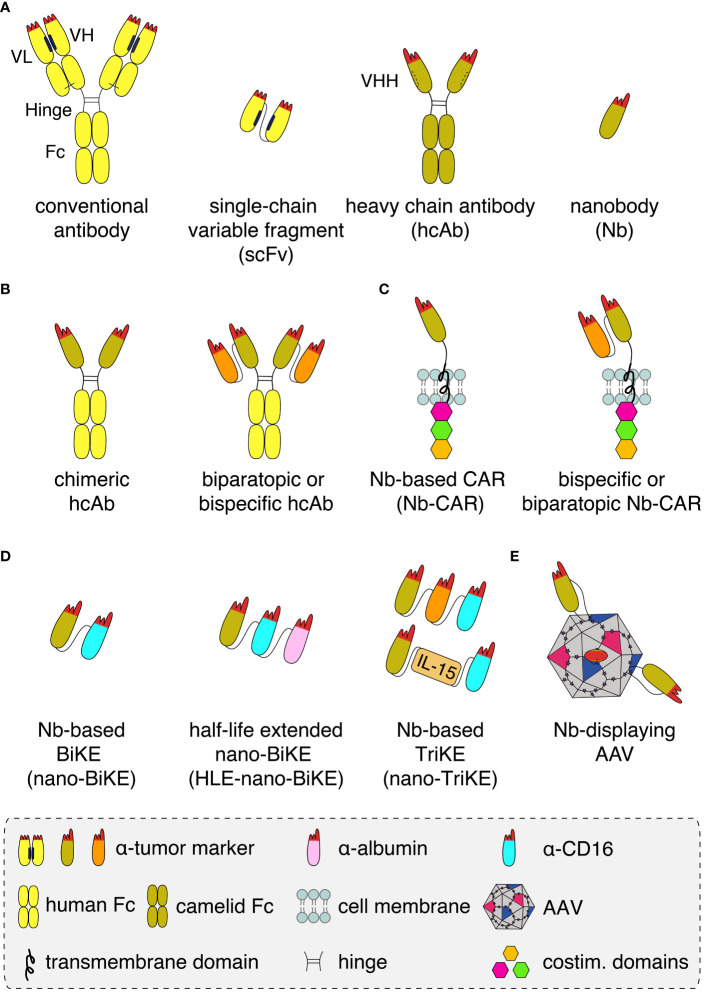
Schematics of nanobody-based biologics **(A)** Conventional antibodies (yellow) contain light and heavy chains that pair *via* hydrophobic regions (black bars) in the framework of the VL and VH domains and by a disulphide bond (light black line) between the CL and CH1 domains. The complementarity-determining regions (red) of VH and VL together form the antigen-binding paratope. A recombinant antigen-binding module, i.e. a single chain variable fragment (scFv), can be derived from a conventional antibody by genetically fusing the VH and VL domains *via* a peptide linker. Heavy chain antibodies (hcAb, olive) derived from llamas lack the light chain and the CH1 domain. A hydrophilic patch (dashed bar) of the VHH domain in place of the corresponding hydrophobic region of a VH domain accounts for the excellent solubility of recombinant VHH domains, i.e. nanobodies (Nb), that allows easy fusion to other proteins and/or Nbs. **(B)** Nanobodies can be converted into hcAbs of any isotype by genetic fusion to hinge, CH2 and CH3 domains, e.g. of human IgG1. Genetic fusion to a second nanobody that recognizes either a second epitope on the same target antigen or a distinct target (orange), results in a biparatopic or a bispecific hcAb, respectively. **(C)** Genetic fusion of one or two nanobodies to a transmembrane domain and to cytosolic costimulatory domains yields a monomeric, biparatopic or bispecific chimeric antigen receptor (CAR) that can be transduced into T cells or NK cells. **(D)** Fusion of one or two tumor marker-specific nanobodies to a nanobody recognizing the Fc-receptor of NK cells (CD16, light blue) yields bispecific or trispecific killer cell engagers (nano-BiKEs or nano-TriKEs). As an alternative to a second tumor-specific nanobody, IL-15 can be fused to a tumor marker-specific nanobody and a CD16-specific nanobody to generate a nano-TriKE. Half-life extension (HLE) of these molecules can be achieved by genetic fusion to an albumin-specific nanobody (magenta). **(E)** Genetic fusion of a membrane protein-specific nanobody into an exposed surface loop of the VP1 capsid protein yields a targeted nanobody-displaying adeno-associated viral vector (AAV) that specifically transduces cells expressing the target antigen.

Nanobodies are highly soluble, single variable immunoglobulin domains derived from heavy chain antibodies (hcAbs) that naturally occur in llamas and other camelids (**Figure 1A**). Due to a splice site mutation, hcAbs lack the CH1 domain of a conventional antibody and do not pair with a light chain (1). Nanobodies contain a hydrophilic region in place of the hydrophobic patch that mediates pairing of VH and VL domains in conventional antibodies (2). Nanobodies thus display a much higher solubility and better developability than individual variable domains or single chain variable fragments (scFv) derived from conventional antibodies. These properties make them particularly suited for specific and efficient targeting of tumor antigens *in vivo* (3, 4).

Nanobodies can be used in a LEGO brick like fashion for the construction of a wide variety of nanobody-based biologics, including biparatopic and bispecific reagents (**Figures 1B–E**). Caplacizumab, the first nanobody-based therapeutic approved for clinical use in 2019, is a genetic fusion of two identical nanobodies against the van Willebrandt factor linked *via* a peptide linker. Caplacizumab is approved for the treatment of acquired thrombotic thrombocytopenic purpura (5). Ciltacel, a nanobody-based therapeutic that recently received fast-track approval by the FDA for the therapy of multiple myeloma, is a biparatopic chimeric antigen receptor (CAR) containing two genetically linked nanobodies fused to the transmembrane and signal transduction modules of different T cell membrane proteins (**Figure 1C**). The two nanobodies recognize non-overlapping epitopes of B cell maturation antigen (BCMA), a cell surface receptor of the TNF superfamily expressed by mature B cells and often overexpressed by multiple myeloma cells (6–9). Remarkably, in order to achieve complete remission, tenfold lower cell numbers are required for CAR-T cells expressing this nanobody-based biparatopic receptor compared to CAR-T cells expressing a single BCMA-specific nanobody. This is thought to reflect the higher affinity, higher stability, and/or lower unspecific off-target binding of the BCMA-specific nanobodies compared to scFvs and conventional VHs (10, 11).

Multiple myeloma is a hematological malignancy that is characterized by the uncontrollable expansion of malignant plasma cells in the bone marrow (12–14). Survival of myeloma patients has improved with new drugs and autologous stem cell transplantation. Despite this progress, the majority of myeloma patients relapse (15–18), underlining the need for more effective treatment options with higher specificity and fewer side effects (15, 19, 20).

CD38 is a type II transmembrane glycoprotein whose extracellular domain is an enzyme that hydrolyzes NAD+. Multiple myeloma cells and other hematological malignancies overexpress CD38. This makes it an interesting target for immunotherapy. Since CD38 is part of the purinergic-signaling cascade that converts NAD+ into immunosuppressive adenosine, the overexpression of CD38 on tumor cells is thought to contribute to an anti-inflammatory tumor microenvironment (21, 22).

Daratumumab and isatuximab are CD38-specific monoclonal antibodies that have been approved for the treatment of multiple myeloma in newly diagnosed and relapsed myeloma patients (23). Genmab isolated daratumumab from human immunoglobulin transgenic mice immunized with CD38 (24, 25). Isatuximab is a chimeric CD38 specific antibody developed by Sanofi from immunized mice (26). Other CD38 specific antibodies such as TAK-079 and MOR202 have also entered clinical trials (23). We have generated CD38-specific nanobodies from immunized llamas (27). Here, we focus on the reformatting of CD38-specific nanobodies into potential immunotherapeutics. The insights gained may be applied also to nanobodies directed against other tumor targets.

## CD38-specific nanobody-based antibody constructs

We have generated nanobodies from llamas using genetic immunization that bind to three independent epitopes of CD38 (27, 28). For both, daratumumab and isatuximab, we have identified nanobodies that bind independent epitopes, i.e. they stain cells that are already covered with daratumumab or isatuximab (27, 29). Such nanobodies are useful for detecting CD38 on the cell surface in patients undergoing daratumumab or isatuximab therapy (30).

We used these CD38-specific nanobodies to generate CD38-specific nanobody-based heavy chain antibodies (hcAbs), chimeric antigen receptor CAR-NK cells, bispecific killer cell engagers (BiKEs), and nanobody-displaying adeno-associated viruses (AAVs) (**Figures 1B–E**) (27, 29, 31–34). Some of these have been radiolabeled and tested as tools for the *in vivo* imaging of multiple myeloma (35). Similarly, the group of Yong Juan Zhao has selected a panel of CD38-specific nanobodies from immunized Bactrian camels (36). One of these, 1G3, was used to generate CD38-specific nanobody-based CAR-T cells (36). Caers et al. radiolabeled some of our nanobodies and the nanobodies by Zhao et al. as a tool for studying fratricide of NK cells under daratumumab therapy (37).

## Nanobody-based heavy chain antibodies (hcAbs)

**Figure 2:**
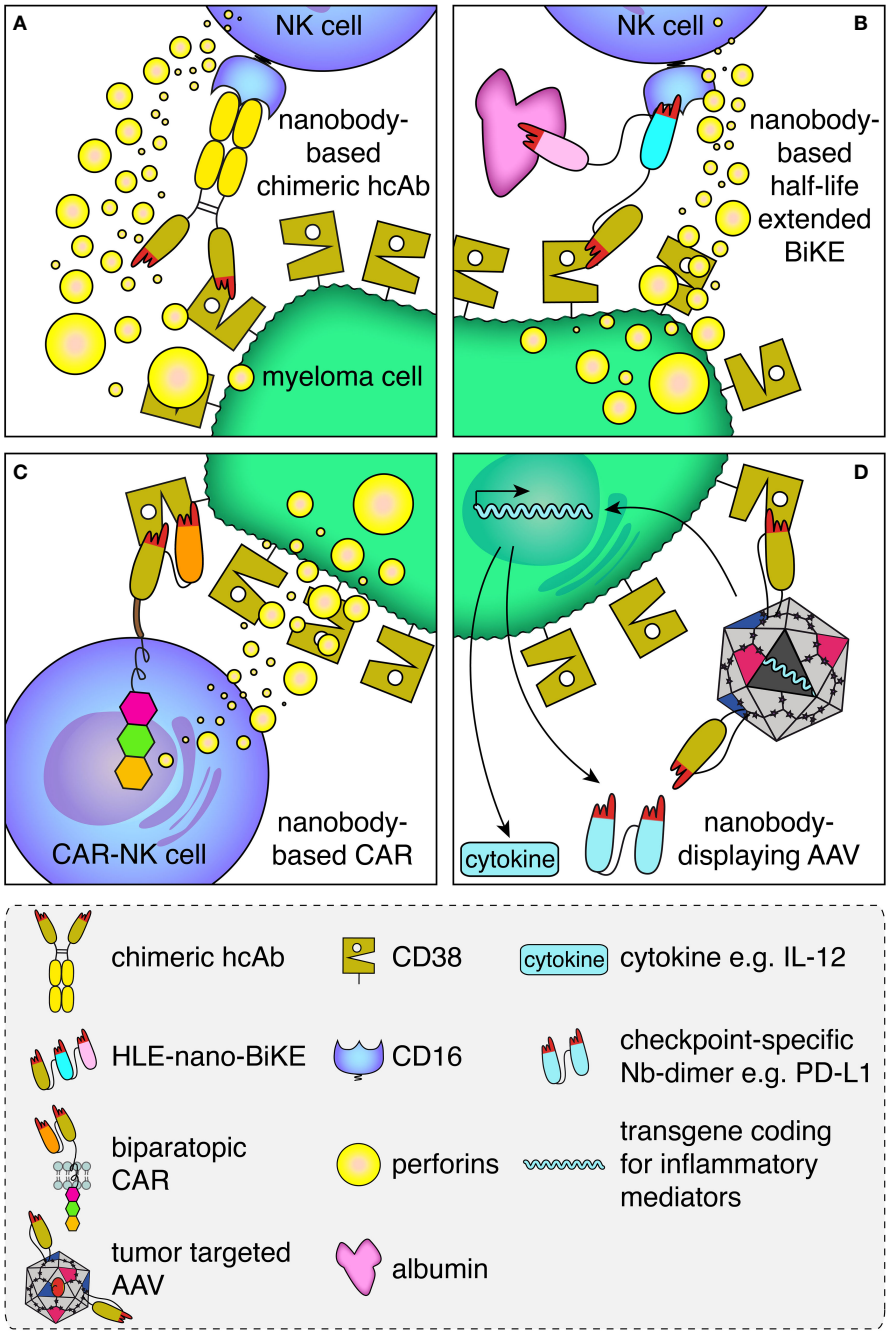
Schematic illustration of the mode of action of the CD38-specific nanobody-based hcAbs, BiKEs, CARs and nanobody-displaying AAVs. **(A)** Antibody-dependent cellular cytotoxicity (ADCC) by an NK cell against a myeloma cell is mediated by a CD38-specific nanobody-based heavy chain antibody. A nanobody-based CD38-specific heavy chain antibody (hcAb) bound to a tumor-antigen (CD38, olive) on the plasma membrane of a multiple myeloma cell is recognized by an Fc-receptor (CD16, blue) of an NK cell. Cross-linking of CD16 on the NK cell induces the release of perforins, which form pores and kill the myeloma cell. **(B)** A half-life extended (HLE)-nano-BIKE crosslinks CD38 on the tumor cell and CD16 on the NK cell, causing release of perforins and killing of the myeloma cell. A third, albumin-specific nanobody (magenta) binds to albumin in the plasma and thereby extends the half-life of the construct by slowing renal filtration. **(C)** An NK cell transduced with a biparatopic nanobody-based chimeric antigen receptor (CAR) binds to CD38. Cross-linking of multiple nano-CARs on the NK cell surface triggers the release of perforins and lysis of the myeloma cell. **(D)** A membrane protein-specific CD38-specific nanobody inserted in the capsid of an AAV mediates specific transduction of tumor cells expressing the cognate target. Expression of the transgene encoding a pro-inflammatory cytokine (e.g. IL-12) and/or a checkpoint blocking nanobody dimer (e.g. α-PDL-1) helps to convert an immunosuppressive into an inflammatory tumor microenvironment.

Binding of therapeutic antibodies to a target cell can induce various effector functions including antibody-dependent cellular cytotoxicity (ADCC), complement dependent cytotoxicity (CDC), antibody-dependent cellular phagocytosis (ADCP), and antibody-induced apoptosis (38). ADCC was shown to be a major mode of action of daratumumab (39, 40). In ADCC, the Fc-part of an antibody bound to the target cell is recognized by the Fc-receptor CD16 on an NK cell (**Figure 2A**). This activates the NK cell to release perforins, which ultimately lyse the target cell by forming pores.

Genetic fusion of a nanobody with the hinge and Fc-domains of human IgG1 generates a chimeric heavy chain antibody (hcAb) (**Figures 1B**, **2A**) (41, 42). We generated such CD38-specific nanobody-based hcAbs and evaluated their potential to induce ADCC and CDC (29, 32). These hcAbs showed potent ADCC toward CD38 expressing tumor cell lines *in vitro* and to multiple myeloma cells from patient bone marrow samples *ex vivo* independent of the bound epitope, but failed to induce CDC of these cells. In a xenograft model, mice treated with the hcAbs showed significantly improved survival. This is consistent with results with the CD38-specific antibody MOR202, which also did not induce CDC *in vitro* but performed well in clinical trials, although not as well as daratumumab (43).

The relative roles of ADCC and CDC in the overall success of therapeutic antibodies in multiple myeloma are as of yet unknown. It is more likely that a combination of effects underlies therapeutic efficacy. However, our results imply that ADCC may be more important than CDC, at least in a xenograft model *in vivo*. The ability of therapeutic antibodies to induce effector functions can be manipulated through directed mutagenesis of specific amino acids in the Fc-part of the antibody. For example, introduction of the single point mutation E345A, a so-called hexabody mutation, into CD38-specific IgG1 hcAbs, conferred the capacity to potently induce CDC (29).

Effective CDC can also be triggered when two hcAbs that bind to different epitopes on CD38 are combined, although neither can trigger CDC independently. This is likely due to cross-linking of CD38 on the tumor cell. Based on this observation, we generated tetravalent biparatopic hcAbs. In these constructs, two different CD38-specific nanobodies connected by a GS linker are genetically fused to the hinge, CH2 and CH3 domain of IgG1, generating tetravalent hcAbs (**Figure 1B**). These hcAbs also triggered effective CDC against CD38-expressing cell lines.

Antibody-mediated modulation of the enzyme function of CD38 may also contribute to changing a “cold” into a “hot” tumor microenvironment (22, 44). Some of our hcAbs, i.e. those that bound to epitope 2, indeed inhibited the GDPR-cyclase activity of CD38, as had been reported also for daratumumab and isatuximab (22, 27). However, none of these antibodies affected the NAD-hydrolase activity, i.e. the main enzymatic activity of CD38.

CD38 is not only expressed by tumor cells, but also by regulatory B and T cells as well as by myeloid suppressor cells and NK cells. Simultaneous targeting of CD38 on regulatory cells might contribute to a less immunosuppressive tumor milieu and to the expansion of effector T cells (38). In contrast, targeting of NK cells, as evidenced by a reduction of CD38-expressing NK cell numbers during daratumumab treatment may contribute to tumor relapse (38). Therefore, it is desirable to design better antibody-constructs that specifically target myeloma cells and spare healthy cells.

To increase the specificity of hcAbs further, the principle of a biparatopic hcAb could be extended to a bispecific hcAb, in which two nanobodies with specificities for two different tumor markers are combined in one construct. This might hamper tumor escape from immunotherapy by antigen loss. By selectively using nanobodies with a low affinity for their target, it might be possible to generate hcAbs that only bind when both target antigens are expressed on the target cell.

In conclusion, hcAbs represent a promising new type of anti-tumor biologic. Our CD38-specific chimeric hcAbs and biparatopic hcAbs showed effectivity *in vitro*, *ex vivo*, and *in vivo*, deserving further verification in clinical trials.

## Nanobody-based bispecific killer cell engagers (BiKEs)

Bispecific engagers such as bispecific T cell engagers (BiTEs) or bispecific killer cell engagers (BiKEs) are designed to recruit cytotoxic immune cells to tumor cells. In a classical BiTE, a tumor antigen specific scFv is linked to an scFv specific for CD3 (part of the T cell receptor complex) *via* a peptide linker (45, 46). Binding of the BiTEs to the cell surface of tumor cells expressing the target antigen, subsequently recruits T cells *via* multivalent binding of the CD3-specific scFvs on the BiTE-coated tumor cell surface. Formation of an immunological synapse between the target cell and the T cell induces activation of the T cell. The activated T cell releases perforins that cause lysis of the tumor cell (46). Similarly, bispecific NK cell engagers (BiKEs) feature a tumor cell-specific scFv linked to an scFv that is specific for CD16 (the high affinity Fc-receptor) on an NK cell in place of the CD3-specific scFv (47).

Nanobodies seem particularly suited for the construction of BiTEs and BiKEs because of their better solubility and reformatability (**Figure 2B**) (2). Several groups have constructed chimeric BiTEs and BiKEs that contain a tumor-antigen-specific nanobody fused to an scFv for targeting either CD16 or CD3 (48–50). Others have constructed BiKEs that contain two nanobodies (51–53). For example, Van Faassen et al. generated a series of nanobody-only BiKEs directed against CD19, HER2 and EGFR that showed good killing efficacy against cells displaying the respective target on the cell surface (54).

The small size of BiTEs and BiKEs, whether composed of scFvs or of nanobodies, lies below the size of the renal filtration barrier, leading to rapid renal excretion. Various strategies have been used to extend the half-life of BiKEs and BiTEs *in vivo*. For example, fusion to hinge and Fc-domain of IgG or to an albumin-specific binding element can impede renal filtration and increase the *in vivo* half-life of BiKEs or BiTEs. Such constructs are called half-life extended (HLE-BiKE) (55–57). Using CD38-, CD16- and albumin-specific nanobodies, we recently developed CD38-specific half-life extended nanobody-based BiKEs (HLE-nano BiKEs) (33). These recruited CD16-expressing NK92 cells to exert potent cytotoxicity against CD38-expressing tumor cell lines *in vitro* and primary multiple myeloma cells *ex vivo*, independent of the epitope bound on CD38. In our preclinical set up, HLE-nano BiKEs induced NK92-mediated cytolysis even more effectively than daratumumab. Biolayer interferometry analyses confirmed simultaneous binding of all three nanobodies to their respective target. Importantly, simultaneous binding of albumin did not interfere with BiKE-mediated killing. Moreover, we encountered no problems with aggregation during production, purification, and concentration of our HLE-nano BiKEs, which in contrast is often observed with scFv-based constructs (58–60).

For BiKEs, the CD16-specific binding element used may influence the effectiveness of the BiKE. A number of CD16-specific nanobodies (54, 61) that show a range of affinities for CD16a could mediate different effectiveness as a binding element in the BiKE. Van Faassen et al. developed a nanobody that not only binds CD16a, which is expressed on NK cells, monocytes, and macrophages, but also CD16b, which is expressed on neutrophils and is important for phagocytosis (54). These authors also investigated the influence of the order of nanobodies in the BiKE and the linker length (54). They found no significant differences in killing between BiKEs containing the same nanobodies with different linker lengths or arrangements of nanobodies.

BiKE-mediated NK cell cytotoxicity and antibody mediated cellular cytotoxicity (ADCC) both activate NK cells *via* CD16. In case of antibodies, the binding affinity of CD16 to the Fc-part of the antibody may influence the effectivity of ADCC (62). Therapeutic antibodies may also be affected by the prevalence of physiological IgG in plasma (63). Thus, antibodies present in serum may already occupy high affinity Fc-receptors on immune cells. To overcome this competition, high levels of therapeutic antibodies or antibodies engineered for increased affinity to CD16 may be needed. Such problems are likely circumvented by BiKEs that contain a nanobody that binds to CD16 independent of its occupancy by IgG. Fusion of an additional tumor-specific binding module or a co-stimulatory interleukin such as IL-15 converts a bispecific engager into a trispecific engager, also called TriKE (45, 64, 65). TriKEs have shown increased efficiency compared to BiKEs (66, 67).

Given their excellent solubility, stability, and their ease of production, we propose further *in vivo* evaluation of nanobody-based BiKEs and TriKEs versus scFv-based bispecific engagers for the therapy of multiple myeloma.

## Nanobody-based chimeric antigen receptors (CARs)

T cells or NK cells equipped with a chimeric antigen receptor (CAR) can bind to tumor antigens independent of their native T cell or Fc-receptor and thereby induce CAR-dependent cellular cytotoxicity (CAR-DCC) (**Figure 2C**). CARs of the first generation featured a binding element such as a tumor marker-specific scFv fused *via* an extracellular linker to a transmembrane domain and a cytosolic immunoreceptor tyrosine-based activation motif (ITAM) of CD3. The introduction of additional ITAMs of other costimulatory receptors such as 41BB, OX40 or CD28 led to the development of second and third generation CARs (68).

In CAR-T therapy, T cells of the patient are genetically modified by transduction with a lentiviral vector to express a tumor marker-specific CAR and are then expanded *ex vivo*. The expanded cells are transplanted back to the patient, often inducing a storm-like release of cytokines due to massive activation of the transplanted T cells. The side effects of this cytokine storm often require management of the patient on an intensive care unit. Another drawback of CAR-T cell-based therapies is the dependence on the quality of the graft (69, 70). Since cells are harvested from already diseased and often heavily pre-treated patients, the fitness and quantity of cells obtained from a patient may be reduced.

NK cell-based CAR cells represent an alternative in this regard. NK cells show fewer side effects during transplantation than T cells (71). In addition, NK cells from established cell cultures or allogeneic transplants show lower graft-versus-host effects (72, 73). This may allow the use of CAR-NK cells as an “off-the-shelf” therapy, since new CAR cells do not have to be individually produced for each patient (74).

Although widely used, scFvs exhibit inherent structural properties that limit their applicability *in vivo*. The VH and VL domains associate *via* a hydrophobic interface that is inherently instable. Dissociation of the domains at this interface may impede expression levels of the CAR on the cell surface. Moreover, the exposed hydrophobic surfaces of the VH and VL domains may account for nonspecific binding to off target cells (11). This may lead to early depletion of CAR-T cells and may result in a decreased success of therapy. The inherent instability of scFv also hampers the construction of CARs composed of two or more scFvs, i.e. bispecific or biparatopic tandem CARs, as the VH and VL of the two scFvs could mismatch.

As in the case of BiTEs and BiKEs, high solubility and easy reformatability render nanobodies particularly suited for the construction of CARs (**Figure 2C**). The introduction of nanobodies as binding domain for these artificial receptors led to the development of CARs with fine-tuned binding properties (11). Moreover, due to their small size, it is possible to insert more than one nanobody into the CAR (10, 75, 76). This gives researchers the opportunity to further refine the specificity and affinity of a nanobody-based CAR. For example, tandem linkage of two nanobodies that bind to independent epitopes of BCMA resulted in BCMA-specific biparatopic CARs with higher affinities for the target and exceptionally high effectivity of the derived CAR-T cells (10, 11).

With respect to CD38, several scFv-based CAR-T and NK cells have been developed (77–79), some of which are currently under clinical evaluation (clinical trials NCT03473496, NCT03464916, NCT04861480. June 2022, clinicaltrials.gov). Some of these were affinity-optimized to more specifically target cells that highly express CD38 rendering a very potent CD38-specific CAR. Recently, we and others have used CD38-specific nanobodies for the construction of nanobody-based CARs (31, 36). The nanobody-based CAR-T cells developed by An et al. displayed significant cytotoxicity and cytokine production *in vitro* as well as *in vivo* in a xenograft mouse model (36). We expressed our nanobody-based CARs on NK92 cells as a basis for “off-the-shelf” CAR-NK cells. These nanobody-based CAR-NK cells also showed potent cytotoxicity against CD38+ cells *in vitro* and *ex vivo*. The effectiveness of the CAR-NK cells was interestingly independent of the epitope on CD38 bound by the nanobody.

Since healthy cells also express CD38, it would be advantageous to develop CAR cells that specifically kill CD38 overexpressing myeloma cells. Conceivably, one could introduce one or more additional nanobodies that recognize another membrane protein expressed on the surface of the myeloma cell such as CD138, CD319 or CD229. Such a bispecific CAR might improve tumor cell binding of CAR-T or CAR-NK cells over binding of healthy cells that only express one of the two target proteins.

Nanobody-based CARs are easier to engineer genetically than scFvs. Nanobody-based CARs can be designed for their affinity, avidity, and specificity through their LEGO brick-like structure. Therefore, nanobody-based CAR cells represent a promising technology to combat multiple myeloma.

## Nanobody-displaying AAV vectors

Adeno-associated viral vectors (AAVs) are well-established tools for gene therapy (80, 81). AAVs are non-replicative, non-enveloped, small ssDNA viruses. Typically the nucleotide sequences coding for the viral capsid and replication genes are replaced by the nucleotide sequence of a gene of interest to be delivered to the transduced target cells. This sequence may encode a fluorescent reporter, a protein or RNA modulator, a toxin, an antibody or nanobody/hcAb, an inflammatory cytokine, a microRNA or a CRISPR/Cas9 targeting module (82–86). However, AAVs often show a broad tropism, hampering delivery of the gene of interest to a specific cell type such as a tumor cell (87). Mutations of and peptide insertions into the capsid proteins have been used to reduce binding of the AAV vector to ubiquitously expressed targets (e.g. glycoproteins) and to increase the selectivity for certain cells and tissues (88). More specific targeting technologies have also been developed to direct the AAV capsid to specific cell surface receptors, e.g. by genetic fusion or chemical ligation of an AAV capsid protein to the ligand of a cell surface receptor (89–91). For example, HER2-specific design ankyrin repeat proteins (DARPins) have been used for the improved transduction of HER2-expressing cells through AAV2 (92).

Here too, the high solubility and easy reformatability could render nanobodies particularly suited as targeting modules for AAVs, e.g. to myeloma cells overexpressing CD38. To this end, we inserted a CD38-specific nanobody into the exposed GH2/GH3 surface loop of the VP1 viral capsid protein of AAV2 (34). Since the N terminus of a nanobody is located close to the antigen-binding paratope while the C terminus is on the opposite side, we used a short linker on the C terminus and a longer linker on the N terminus to allow an upright orientation of the nanobody on the capsid surface (**Figure 2D**). All CD38-specific nanobodies tested as well as several other nanobodies targeting structurally distinct membrane proteins were successfully inserted in this way. This suggests that this strategy likely is suited for a broad range of nanobodies and target membrane proteins. For each of the inserted nanobodies, we observed a 10- to 500-fold enhanced AAV transduction of target-transfected HEK cells and other target-protein-expressing cells. Remarkably, AAVs displaying a CD38-specific nanobody also specifically transduced CD38-expressing myeloma cells in primary bone marrow samples of multiple myeloma patients. We analyzed the transduction efficiency *via* a GFP reporter as transgene - for future applications the nanobody-displaying AAVs could encode inflammatory mediators, e.g. pro-inflammatory cytokines or nanobody dimers that block immune checkpoint proteins like PD-L1 (**Figure 2D**).

In conclusion we propose that nanobodies represent a means to combine the benefits of AAV-mediated gene transfer with specific targeting of myeloma cells. Nanobody-displaying AAVs pave the way for a wide range of applications as vectors for delivery of immunomodulatory substances such as cytokines or checkpoint inhibitors to the tumor microenvironment.

## Discussion

Nanobodies display an inherent robustness and high solubility that has been shaped by millions of years of evolution in the camelid lineages. In contrast, single variable immunoglobulin domains (VH or VL) derived from conventional human antibodies often show a tendency to aggregate in the absence of its natural partner (93, 94). This is attributed to hydrophobic surface patches in the framework regions where VH and VL naturally associate. In the context of a natural antibody the pairing and covalent linkage of the adjacent CH1 and CL domains additionally stabilizes the association of VH and VL domains. Conversely, the pairing of VH and VL domains generally is less stable in the context of a recombinant scFv, where a VH and VL domains are genetically fused *via* a peptide linker. These inherent structural features likely account for the much better developability of nanobody-based biologics than of scFv-based biologics.

Two nanobody-based biologics have been granted FDA approval for clinical use: Caplacizumab, a homodimer of two identical van Willebrandt factor-specific nanobodies connected by a peptide linker and Ciltacel a chimeric antigen receptor composed of a heterodimer of two different BCMA-specific nanobodies that target non-overlapping epitopes of BCMA. The size of Caplacizumab is much smaller than that of a conventional antibody and lies below the size of the renal filtration barrier. Consequently, Caplacizumab exhibits a short half-life *in vivo* and is intended for short-term treatment of blood clotting disorders. The BCMA-specific biparatopic nanobody of Ciltacel, in contrast, is expressed as a cell surface receptor on transduced T-cells and is intended for long-term treatment of BCMA-expressing multiple myeloma.

The entry of nanobodies into the clinic likely has been slowed by concerns regarding the potential immunogenicity of the camelid VHH domain in human patients. In general, immunogenicity of variable immunoglobulin domains is much less of a concern than that of constant immunoglobulin domains and other proteins. Humans carry ~50-150 distinct VH and VL encoding gene fragments. Somatic hypermutation during natural immune responses generates millions of variants of the germline VH and VL domains. During pregnancy, such variants are transferred across the placenta from the mother to the fetus. This likely accounts for the high natural tolerance against VH and VL domains of other species.

To mitigate immunogenicity concerns, framework residues in nanobodies intended for clinical use in humans, usually are “humanized” (95). This involves substitutions of amino acid residues on the surface of the VHH domain by corresponding residues commonly found in human VH domains. Owing to the natural similarity of VHH domains to human VH3 domains, this is often possible without compromising the stability or functionality of the nanobody, perhaps with the exception of the hydrophilic residues at the former interface to the VL domain.

The astounding efficacy of the nanobody-based Ciltacel (96) compared to CARs based on scFvs raises a number of questions that need be addressed in future studies. Is a biparatopic CAR generally more efficient than a monovalent CAR? Do the epitopes recognized by the nanobodies influence the efficacy? Could the efficacy of a CAR be increased by combining two nanobodies directed against distinct cell surface receptors such as CD38 and BCMA that are co-expressed by the target cell? It also remains to be seen whether the astounding efficacy of the nanobody-based CARs can be reached also with nanobody-based CARs targeting other cell surface proteins (11, 97).

In this review we focused on the applicability of CD38-specific nanobody-based constructs for the treatment of multiple myeloma. Targeting CD38 with such constructs could also be a promising alternative for the treatment of other CD38 positive malignancies. CD38 is also often highly expressed in other hematological malignancies, e.g. acute myeloid leukemia (AML) (98), and chronic lymphocytic leukemia (CLL) (99). Moreover, CD38 over- expression by cancer-associated fibroblast has been suggested to promote a pro-tumoral activity in melanoma (100). Expression of CD38 by lung cancer and other solid tumors may also contribute to an oncogenic tumor microenvironment by promoting the enzymatic conversion of NAD+ to immunosuppressive adenosine (101, 102). CD38-specific nanobody-based constructs that inhibit its enzyme activity would be targeting the “Achilles heel” of such tumors.

Most tumors are heterogeneous, and tumor cells that do not express or loose expression of the target membrane protein of a therapeutic antibody would have a growth advantage (103, 104). In such cases, simultaneous targeting of two or more tumor cell membrane proteins would impede escape and lower the rate of recurrence (105). The specificity of nanobody-based constructs for tumor cells might be enhanced by simultaneous targeting of multiple tumor-specific antigens through integration of additional nanobodies into the construct. The small size of nanobodies and ease of reformatability facilitate the construction of bispecific hcAbs, BiKEs, CARs, and nanobody-displaying AAV vectors (29, 33, 53).

CD38 is also expressed by in multiple different cell types of innate and acquired immunity, including both, tumor promoting cells and cells with cytotoxic activity (21, 23). Targeting of regulatory T cells and B cells, and myeloid derived suppressor cells would be beneficial, whereas targeting of NK cells and activated cytotoxic T cells would be counter-productive (78, 106, 107). As in case of tumor heterogeneity, by combining nanobodies with low affinity for two targets that are co-expressed by tumor cells but not by NK or T cells, e.g. CD38 and BCMA, it might be possible to specifically deplete only cells expressing both target antigens and thus to develop nanobody-based antibody constructs with lower off-target effects.

The results of our own studies with CD38-specific nanobodies illustrate the utility of nanobody-based heavy chain antibodies, BiKEs, CARs and nanobody-displaying AAV vectors to specifically and effectively target CD38-expressing myeloma cells. The promising results of our preclinical studies warrant further clinical studies to evaluate the potential of these CD38-specific nanobody-based constructs for treatment of multiple myeloma.

## Author contributions

All authors conceived the topic, PB and FK-N acquired funding, JH and FK-N wrote the manuscript. All authors contributed to the article and approved the submitted version.

## Funding

This work was supported by grants from the Deutsche Forschungsgemeinschaft to PB (BA 5893/7) and FK-N (No310/16, SFB1328- Z02, BMBF-COMMUTE).

## Acknowledgments

We thank Fabienne Seyfried, Birte Albrecht, and Josephine Gebhardt for excellent technical assistance.

## Conflict of interest

PB and FK-N are co-inventors on a patent application on CD38-specific nanobodies. AM and FK-N are co-inventors on a patent application on nanobody-displaying AAV vectors. FK-N receives a share of antibody and protein sales *via* MediGate GmbH, a wholly owned subsidiary of the University Medical Center Hamburg-Eppendorf.

The remaining author declares that the research was conducted in the absence of any commercial or financial relationships that could be construed as a potential conflict of interest.

MediGate GmbH was not involved in the study design, collection, analysis, interpretation of data, the writing of this article, or the decision to submit it for publication.

## Publisher’s note

All claims expressed in this article are solely those of the authors and do not necessarily represent those of their affiliated organizations, or those of the publisher, the editors and the reviewers. Any product that may be evaluated in this article, or claim that may be made by its manufacturer, is not guaranteed or endorsed by the publisher.
